# Transfer Learning-Based Autosegmentation of Primary Tumor Volumes of Glioblastomas Using Preoperative MRI for Radiotherapy Treatment

**DOI:** 10.3389/fonc.2022.856346

**Published:** 2022-04-14

**Authors:** Suqing Tian, Cuiying Wang, Ruiping Zhang, Zhuojie Dai, Lecheng Jia, Wei Zhang, Junjie Wang, Yinglong Liu

**Affiliations:** ^1^Department of Radiation Oncology, Peking University Third Hospital, Beijing, China; ^2^Department of Oncology, Hainan Third People’s Hospital, Sanya, China; ^3^Department of Radiation Oncology, The First Hospital of Tsinghua University, Beijing, China; ^4^United Imaging Research Institute of Intelligent Imaging, Beijing, China; ^5^Shenzhen United Imaging Research Institute of Innovative Medical Equipment, Shenzhen, China; ^6^Shanghai United Imaging Healthcare Co.Ltd., Shanghai, China

**Keywords:** glioblastoma, autosegmentation, deep learning, transfer learning, radiotherapy treatment

## Abstract

**Objectives:**

Glioblastoma is the most common primary malignant brain tumor in adults and can be treated with radiation therapy. However, tumor target contouring for head radiation therapy is labor-intensive and highly dependent on the experience of the radiation oncologist. Recently, autosegmentation of the tumor target has been playing an increasingly important role in the development of radiotherapy plans. Therefore, we established a deep learning model and improved its performance in autosegmenting and contouring the primary gross tumor volume (GTV) of glioblastomas through transfer learning.

**Methods:**

The preoperative MRI data of 20 patients with glioblastomas were collected from our department (ST) and split into a training set and testing set. We fine-tuned a deep learning model for autosegmentation of the hippocampus on separate MRI scans (RZ) through transfer learning and trained this deep learning model directly using the training set. Finally, we evaluated the performance of both trained models in autosegmenting glioblastomas using the testing set.

**Results:**

The fine-tuned model converged within 20 epochs, compared to over 50 epochs for the model trained directly by the same training set, and demonstrated better autosegmentation performance [Dice similarity coefficient (DSC) 0.9404 ± 0.0117, 95% Hausdorff distance (95HD) 1.8107 mm ±0.3964*mm*, average surface distance (ASD) 0.6003 mm ±0.1287mm] than the model trained directly (DSC 0.9158±0.0178, 95HD 2.5761 mm ± 0.5365mm, ASD 0.7579 mm ± 0.1468mm) with the same test set. The DSC, 95HD, and ASD values of the two models were significantly different (*P*<0.05).

**Conclusion:**

A model developed with semisupervised transfer learning and trained on independent data achieved good performance in autosegmenting glioblastoma. The autosegmented volume of glioblastomas is sufficiently accurate for radiotherapy treatment, which could have a positive impact on tumor control and patient survival.

## Introduction

Glioblastoma is the most common primary malignant brain tumor in adults (1). At present, the standard treatment for this disease is combination therapy, including postoperative radiotherapy and adjuvant chemotherapy after the initial surgery. Intensity-modulated radiotherapy (IMRT) is a commonly used method for delivering radiotherapy to glioblastomas. An accurate radiotherapy plan is required to ensure accurate patient treatment (2). The delineation of brain tumor targets and other brain tissue structure areas from multimodal MRI sequences can provide important information for the radiotherapy plan. Traditionally, the manual contouring of these areas is time-consuming and dependent on the experience of the doctors.

The implementation of deep learning has resulted in the development of new ideas for the automatic and accurate delineation of brain tumors (3). Deep learning approaches through convolutional neural networks (CNNs) have been proposed for glioblastoma segmentation (4–6). For example, Yi et al. (4) developed a framework of three-dimensional (3D) fully CNN models for glioblastoma segmentation from multimodality MRI data and achieved a Dice score of 0.89 in whole tumor glioblastoma segmentationon the segmentation dataset of the Brain Tumor Image Segmentation Challenge (BRATS) with 274 tumor samples.

Recently, transfer learning has found multiple applications in brain MRI (7). Transfer learning allows the reuse of a pretrained model to solve a related target problem, potentially yielding better results from fine-tuning pretrained CNNs than training CNNs from scratch (8). In this work, we provide a deep learning model for the autosegmentation of the gross tumor volume (GTV) of glioma. A deep learning model trained for hippocampus autosegmentation was fine-tuned and trained using a limited MR dataset of 20 glioblastoma patients. This approach is expected to serve as a basis for accurate radiotherapy dose calculation and optimization in the development of a high-quality radiotherapy plan (9).

## Materials and Methods

### Patient Characteristics

We retrospectively collected the MRI scans and medical records of patients with histologically proven glioblastomas from a single institute (Department of Radiation Oncology of Peking University Third Hospital). The MRI examinations were performed with preoperative contrast-enhanced T1-weighted sequences. Details of the MRI characteristics are shown in **Table 1**. The MRI dataset consisted of GTVs of high-grade gliomas (HGGs) of 20 patients, which was then randomly split into three cohorts: 16 patients as the training set (including 4 patients as the validation set) for training an autosegmentation model and optimization of hyperparameters during model training and 4 patients as the test set for evaluating the performance of the trained model.

**Table 1:** Characteristics of MRI.

Cancer	Glioblastoma
Tumor	Gross tumor volume
Grade	High-grade gliomas
Modality	contrast-enhanced T1-weighted imaging
Quantity	20 patients
Resolution	(144~176) × 256 × 256
Spacing [mm^3^]	[1,1,1]

### Gross Tumor Volume Contours by Human Experts

**Figure 1:**
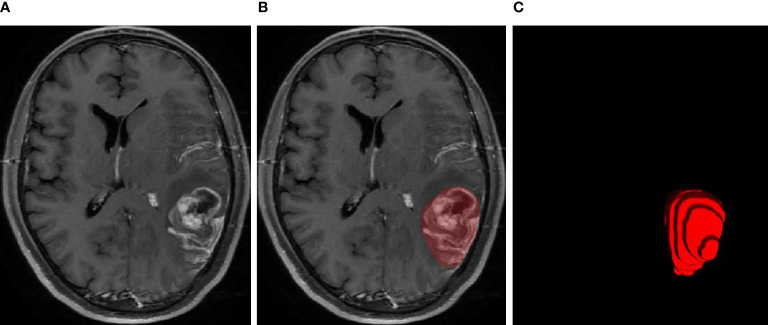
MRI examination of the glioblastoma **(A)**, GTV contours delineated by human experts **(B)**, and 3D diagram corresponding to the GTV contours **(C)**.

The MRI examinations of the 20 patients were assigned to two expert radiation oncologists (ST and ZD, both with more than 15 years of experience in radiotherapy treatment of head and neck tumors) to delineate the ground-truth GTVs *via* consensus. A third radiologist (CW, with more than 20 years of experience) specializing in radiation oncology was consulted in cases of disagreement. A diagram of the GTV contours delineated by the human experts is presented in **Figure 1**.

### Image Processing

#### Preprocessing

All MRI sequences were cropped to only include regions of non-zero value to reduce the size of the network input and thereby reduce the computational load of the network (10). To enable our network to properly learn spatial semantics, all MRI sequences were resampled to the median voxel spacing of the dataset, where third-order spline interpolation was used for the images of all MRI scans and nearest-neighbor interpolation for their corresponding contours. All images were additionally normalized by simple Z score normalization for the individual patients (11).

#### Augmentation

To overcome the overfitting problem caused by training a deep network with limited data, we adopted a number of real-time data enhancement techniques, such as random flip, random zoom, random elastic deformation, gamma adjustment, and mirroring, to increase the diversity of the data.

### Architecture of the Deep Convolution Neural Network

U-Net is a popular encoder-decoder network (11, 12) that has been widely used in semantic segmentation fields. Its encoder part works similarly to a traditional classification CNN in that it successively aggregates semantic information at the expense of spatial information. Its decoder receives semantic information from the bottom layer of the encoder and recombines it with higher-resolution feature maps obtained directly from the encoder through skip connections (13) to recover the spatial information missing in the encoder.

**Figure 2:**
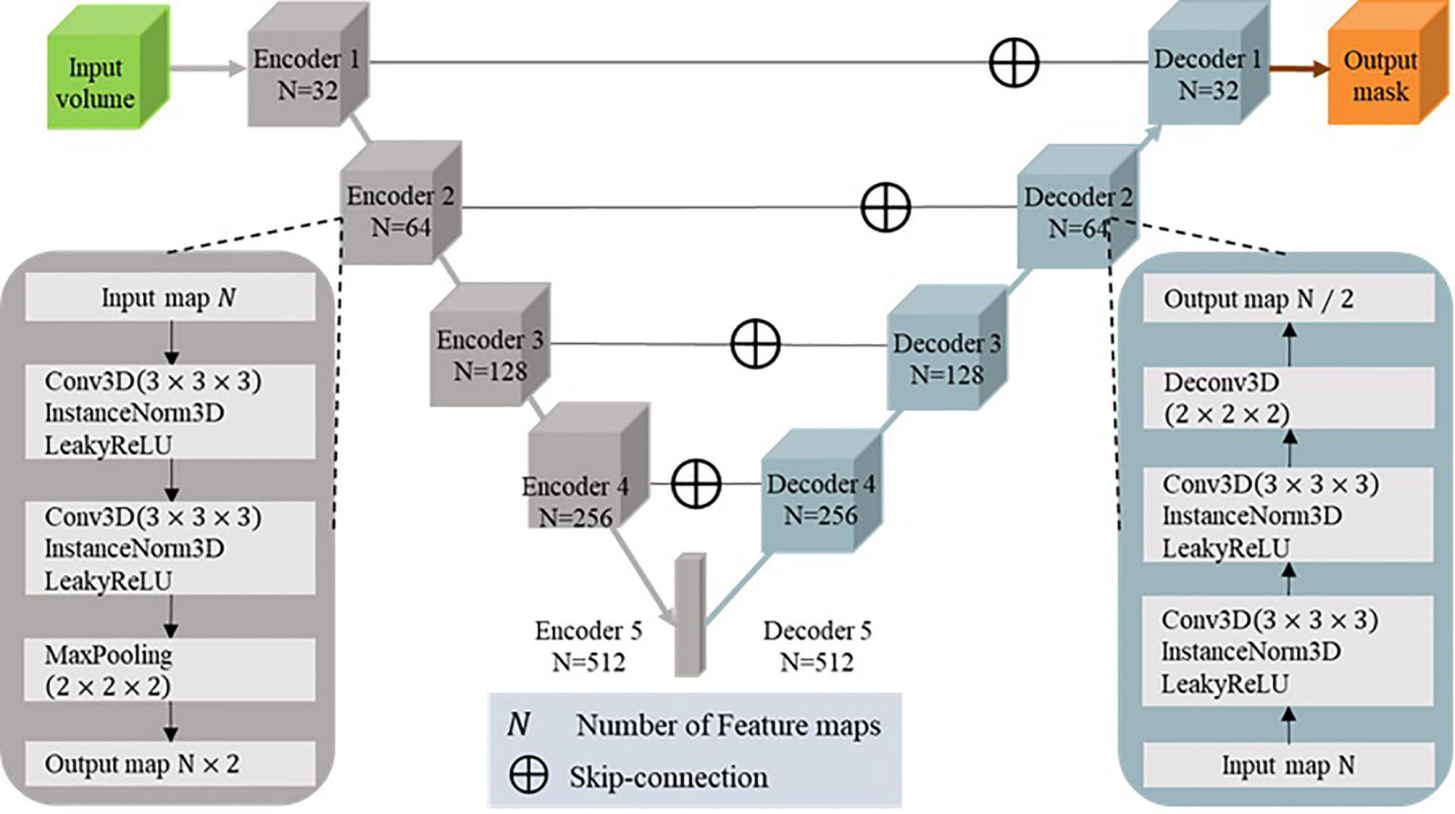
Architecture of the segmentation network.

Since 3D CNNs have demonstrated high effectiveness in aggregating valuable information in the context of 3D medical images (14), we implemented a 3D deep CNN to extract representative features for complicated GTVs based on the MRI sequences. Our network is based on the architecture of 3D U-Net (15), with 5 encoders and 5 decoders. In each encoder and decoder, we designed a couple of convolutional layers with a 3×3×3 convolution kernel to extract the feature of the image, each convolutional layer followed by the LeakyReLU (negative slope 1*e*^–2^) and the instance normalization (16) with a dropout of 0.5, which respectively replaced the more common ReLU activation function and batch normalization in the popular deep learning model. We used the 2×2×2 max pooling to create a downsampled feature map in each encoder; conversely, we used the 2×2×2 deconvolution kernel to create an upsampled feature map in each decoder. The layers in the encoders were skip connected and concatenated with layers in the corresponding decoders to use fine-grained details learned in the encoders to construct the feature maps in the decoders. The detailed architecture of our network is shown in **Figure 2**.

### Objective Loss

Due to the limited available Graphics Processing Unit (GPU) memory, we slid and cropped smaller image patches from the original images as the input of the segmentation network. Although this patch-based training method limits the field of view of the model and is unable to collect sufficient contextual information, the impact on small target segmentation is minimal.

The objective loss *L* of the segmentation network is the weighted sum of Dice loss *L_dice_* and cross-entropy loss *L_CE_*:


$$L=\alpha_{1}L_{dice}+\beta_{1}L_{CE}$$

Here, the weights *α*_1_ and *β*_1_ were set to 0.4 and 0.6, respectively.


$$L_{dice}=-\frac{2}{C}\sum_{k\in C}\frac{\sum_{i\in N}G_{i}^{k}P_{i}^{k}}{\sum_{i\in N}G_{i}^{k}+\sum_{i\in N}P_{i}^{k}}$$

$$L_{CE}=1-\frac{1}{N}\sum_{i\in N}\sum_{k\in C}G_{i}^{k}\log P_{i}^{k}$$

where *C* is the number of divided categories, *N* is the number of voxels in each patch sample in the training set, and $G_{i}^{k}$ and $P_{i}^{k}$ are the contour corresponding to the *i^th^* voxel of the *k^th^* category and the probability output of the model prediction, respectively.

### Experiments and Evaluation

#### Model Implementation

We used PyTorch 1.6 to build 3D U-Net on Ubuntu 18.04 and trained the model framework on an NVIDIA Tesla V100. When training the model, the model input patch size was 32 × 256 × 256, the batch size was 2, the optimizer was RMSprop, the initial learning rate was 0.001, the gradient descent strategy was stochastic gradient descent (SGD) with momentum (0.9), and the maximum number of training rounds (epochs) was 150. In addition, due to the limited amount of collected data, we did not divide the test set separately but adopted an 8:2 dataset division method. For the segmentation results of each patch of the model, we used Gaussian fusion to obtain the full-resolution segmentation result for each class, which was postprocessed with the largest connected component as the final segmentation result.

#### Model Evaluation

We calculated the Dice similarity coefficient (DSC), 95% Hausdorff distance (95HD), and average surface distance (ASD) between the GTVs segmented automatically by the model and the corresponding manual annotations as quantitative assessments of the accuracy of the model segmentation. The DSC is defined as:


$$DSC=\frac{2|P\cap G|}{\left|P|+|G\right|}$$

where *P* is the automatically segmented contour, *G* is the ground-truth contour. DSC is an indicative degree of similarity for agreement, which measures the spatial overlap between the automatic segmentation and the ground-truth segmentation. The 95HD is defined as:


$$d_{H}(P,G)=\max\{d_{PG},d_{GP}\}=\max\left\{\underset{x\in X}{\max}\underset{y\in Y}{\min}d(p,g),\underset{y\in Y}{\max}\underset{x\in X}{\min}d(p,g)\right\}$$

DSC is more sensitive to the inner filling of the segmented contour, while Hausdorff distance (HD) is more sensitive to the boundary of the segmented contour. The 95%HD is similar to maximum HD. However, it is based on the calculation of the 95th percentile of the distances between boundary points in *P* and *G*. The purpose of using this metric is to eliminate the impact of a very small subset of the outliers. The ASD is defined as:


$$\text{ASD}=\frac{1}{\left|S(P)|+|S(G)\right|}\left(\sum_{p\in S(P)}\underset{g\in S(g)}{\min}||p-g||\right)+\sum_{g\in S(G)}\underset{p\in S(P)}{\min}||g-p||$$

where *S*(*P* and *S*(*G*) denote the point set of automatic segmentation pixels and ground-truth pixels, respectively. The most consistent segmentation result can be obtained when ASD equals 0.

#### Model Fine-Tuning

Transfer learning is a process by which existing models are reused to solve a new challenge, usually the problem of overfitting due to data scarcity (17). Given the limited size of the dataset delineated by our human experts and the fact that our modified 3D U-Net is a kind of supervised learning method that works well depending on the severity of the big data, we applied transfer learning to this work to fine-tune the glioblastoma segmentation model.

To apply transfer learning in this work, the 3D U-Net was trained to autosegment the hippocampus with the data from 50 patients with T1C glioblastomas (spacing[mm]: 0.5×0.36×0.36, resolution: (327~364) ×640×640). This hippocampal dataset was collected from a single institute (The First Hospital of Tsinghua University) for the radiotherapy treatment of brain metastases. All contours of the hippocampus were delineated by two expert radiation oncologists (ZD, with more than 15 years of experience in radiotherapy treatment of head and neck tumors; RZ, with more than 20 years of experience in radiotherapy treatment) according to the results of the RTOG0933 trial and then cross-checked and revised.

**Figure 3:**
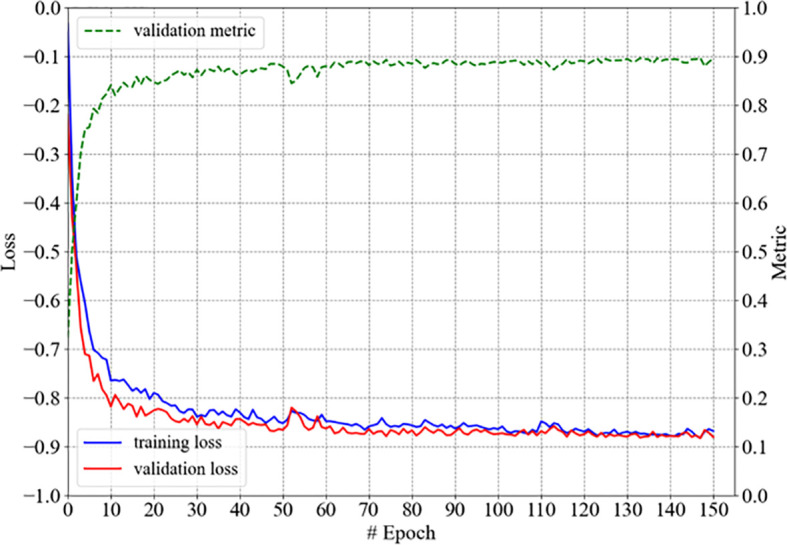
The training process of model-hippo: the loss is the objective loss *L* and the metric is DSC.

Our 3D U-Net for the autosegmentation of the hippocampus was trained with the hippocampus data from 40 patients, converged within 150 epochs, and was denoted as model-hippo; the training process is shown in **Figure 3**. The model achieved a DSC of 0.897 (±0.011) for the left hippocampus and 0.895 (±0.019) for the right hippocampus with the test set (10 cases).

**Figure 4:**
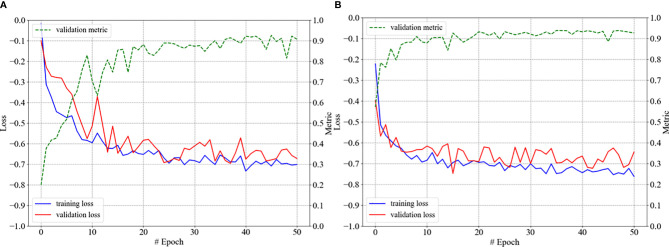
The training processes of model-glioma **(A)** and model-glioma-TL **(B)**.

The model for autosegmenting the GTV of glioblastomas was trained as follows. First, the parameters of our 3D U-Net were randomly initialized, and the model was trained simply with 50 epochs on the training set of gliomas, as illustrated in **Figure 4A**; the model thus developed was denoted as model-glioma. Second, transfer learning was applied to fine-tune model-glioma. Specifically, instead of random parameter initialization, the network parameters of model-hippo were used as the initial parameters of model-glioma, and the resulting model was then trained for 50 epochs using the same training set for training model-glioma. This model was denoted as model-glioma-TL, where TL refers to transfer learning. The corresponding training process is shown in **Figure 4B**.

As shown in **Figure 4**, we found that model-glioma-TL converged faster than model-glioma did within the same 50 epochs. The validation metric reached 0.9 within 10 epochs for model-glioma-TL but within 40 epochs for model-glioma. Moreover, the validation metric of model-glioma-TL on the final epoch was greater than that of model-glioma.

## Results

**Figure 5:**
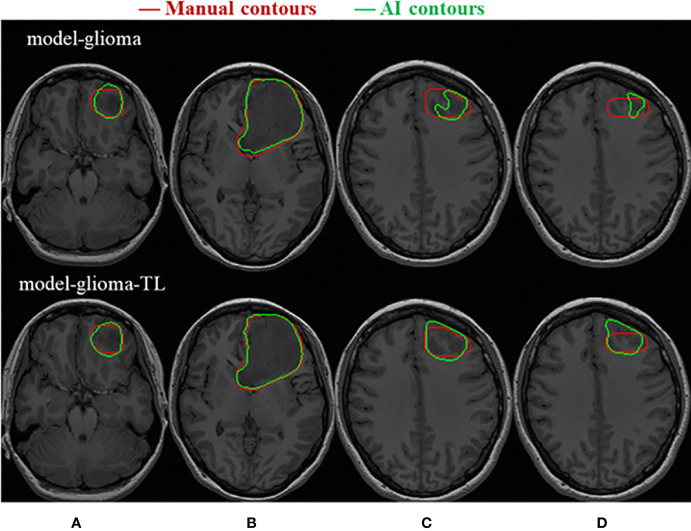
Visualization of the test samples for the two models. The performance of model-glioma-TL was superior to that of model-glioma in the autosegmentation of glioblastoma GTVs, especially in the recognition of the small GTV in the upper and lower MRI slices **(A, D)** and the boundary delineation of the GTV contours in the intermediate MRI slices **(B, C)**.

**Figure 6:**
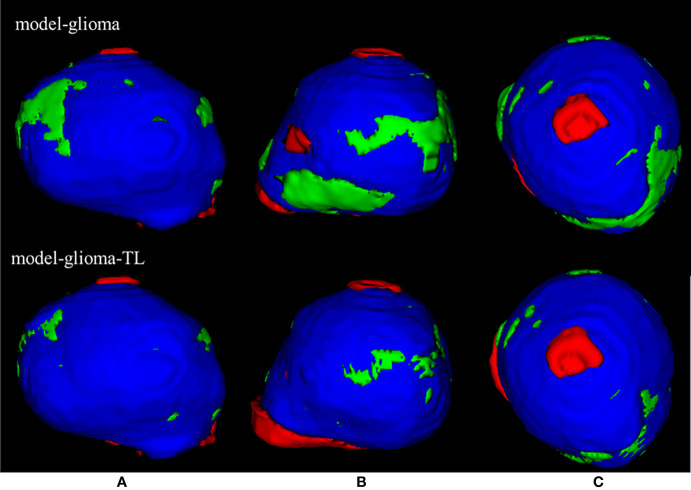
| The 3D visualization of the test samples for the two models. The red region is the individual contouring by human experts, the green region is the individual contouring by AI models, and the blue region is the mutual contouring combined with human experts and AI models. The contouring by model-glioma-TL coincided with the contouring by human experts better than that of model-glioma in three different profiles of the test sample **(A–C)**. And the mean absolute percentage error of model-glioma-TL in the autosegmentation of glioblastoma GTVs with the same test set was 2.58% and superior to 4.74% of modelgli.

Two sets of experiments (model-glioma and model-glioma-TL) were conducted to verify the effectiveness of transfer learning on training with small sample data and to evaluate the performance of the two models by the DSC, 95HD, and ASD metrics. The evaluation metrics of two sets of experiments with the same test set, including the mean, standard deviation (SD), and P value for the T test (two-tailed), are presented in **Table 2**. We found that model-glioma-TL significantly outperformed model-glioma in these terms [DSC 0.9404 & 0.9158, 95HD 1.8107mm & 2.5761mm, ASD 0.6003mm & 0.7579mm (and P<0.05). The autosegmentation results of a test sample are visualized in **Figure 5** and its 3D morphology are visualized in **Figure 6**.

**Table 2:** Results for autosegmentation of the GTVs of glioblastomas.

Model	Model-glioma		Model-glioma-TL		*P* value
	Mean	SD	Mean	SD	
DSC	0.9158	0.0178	0.9404	0.0117	0.0404
95HD [mm]	2.5761	0.5365	1.8107	0.3964	0.0275
ASD [mm]	0.7579	0.1468	0.6003	0.1287	0.0182

## Discussion

The rapid development of modern radiotherapy technology has resulted in more abundant relevant multimodal medical imaging information (18). Since a considerable amount of time is necessary to manually contour MRI slices and the segmentation results of artificial tumors often depend on the doctor’s prior knowledge and work experience, the final target volume results can be variable (19, 20). Therefore, deep learning technology combined with MRI can help improve the accuracy of tumor target delineation and reduce differences caused by subjective factors (10). Additionally, it can help doctors efficiently and practically complete their tumor target area delineation tasks (21, 22).

Gliomas are the most common primary brain tumors and seriously endanger human health (23). Therefore, segmentation of the images of brain tumors has become a popular research topic (24, 25). In recent years, brain tumor image segmentation methods have undergone continuous improvement, transitioning from manual to half-motion and automatic segmentation techniques (22, 26). In the present study, we have demonstrated that the performance of the transfer learning approach is comparable to the models trained through 3D CNN (4, 6), but a much smaller dataset and fewer epochs are required. Indeed, this method should be further evaluated using larger datasets such as BRATS.

In conclusion, transfer learning is feasible and effective in training models for accurate and consistent glioblastoma autosegmentation. This is more crucial for a radiation oncology department that is willing to implement deep learning with limited number of clinical cases.

In this work, the artificial intelligence (AI) algorithm based on transfer learning has achieved good results for the autosegmentation of glioblastoma GTV; however, there are still several issues that need to be cleared. 1) The location and boundary of glioblastoma GTV not only need to consider the enhanced area of contrast-enhanced T1-weighted imaging and the abnormal area of T2 FLAIR in clinical practice, perhaps the autosegmentation of the glioblastoma tumor using multimodality MRI is more satisfying for clinical practice (27). 2) Scanners from different manufacturers or different scanning protocols often result in medical imaging with different voxel spacing and resolution, as well as image quality and style. These differences are especially pronounced for MRI. Additionally, different tumor delineation styles come from the subjectivity of different doctors; these various differences seriously affect the generalizability of the AI algorithms. Therefore, further study using the data from multiple centers is an important topic. 3) We confirmed that transfer learning can significantly improve the automatic segmentation of the glioblastoma GTV in this work; however, some organs or tumor target areas, such as the optic chiasm, have a similar X-shape, while the brain stem has a similar apple shape (28, 29), and it needs to be judged by combining different medical imaging procedures and the rich medical prior knowledge of professional doctors (30, 31). How to incorporate such shape and prior knowledge and medical prior knowledge into the AI model to further improve the generalizability and generalization of AI algorithms still is an open problem.

## Data Availability Statement

The original contributions presented in the study are included in the article/supplementary materials. Further inquiries can be directed to the corresponding authors.

## Author Contributions

Conceptualization, ST and CW. Methodology, ST and YL. Software, YL and LJ. Validation, ST, CW, RZ and ZD. Formal analysis, ST and ZD. Investigation, ST and CW. Resources, ST and JW. Data curation, ST, JW, ZD, and YL. Writing—original draft preparation, ST and YL. Writing—review and editing, JW, CW and RZ. Visualization, YL. Supervision, WZ. Project administration, and WZ. ST and CW contributed equally to this work, so they are listed as co-first authors. All authors contributed to the article and approved the submitted version..

## Conflict of Interest

WZ is employed by Shanghai United Imaging Healthcare Co., Ltd.

The remaining authors declare that the research was conducted in the absence of any commercial or financial relationships that could be construed as a potential conflict of interest.

## Publisher’s Note

All claims expressed in this article are solely those of the authors and do not necessarily represent those of their affiliated organizations, or those of the publisher, the editors and the reviewers. Any product that may be evaluated in this article, or claim that may be made by its manufacturer, is not guaranteed or endorsed by the publisher.
